# Enrichment Environment Positively Influences Depression- and Anxiety-Like Behavior in Serotonin Transporter Knockout Rats through the Modulation of Neuroplasticity, Spine, and GABAergic Markers

**DOI:** 10.3390/genes11111248

**Published:** 2020-10-23

**Authors:** Giulia Sbrini, Paola Brivio, Kari Bosch, Judith Regina Homberg, Francesca Calabrese

**Affiliations:** 1 Department of Pharmacological and Biomolecular Sciences, Università Degli Studi di Milano, 20133 Milan, Italy; giulia.sbrini@unimi.it (G.S.); paola.brivio@unimi.it (P.B.); 2 Department of Cognitive Neuroscience, Donders Institute for Brain, Cognition, and Behavior, Radboud University Medical Center, 6500 HB Nijmegen, The Netherlands; Kari.Bosch@radboudumc.nl (K.B.); Judith.Homberg@radboudumc.nl (J.R.H.)

**Keywords:** serotonin transporter, SERT, Bdnf, spine, GABA, enriched environment

## Abstract

The serotonin transporter (5-HTT in humans, SERT in rodents) is the main regulator of serotonergic transmission in the brain. The short allelic variant of the 5-HTT gene is in humans associated with psychopathologies and may enhance the vulnerability to develop depression after exposure to stressful events. Interestingly, the short allele also increases the sensitivity to a positive environment, which may buffer the vulnerability to depression. Since this polymorphism does not exist in rodents, male SERT knockout (SERT^−/−^) rats were tested to explore the molecular mechanisms based on this increased predisposition. This article investigates the influences of a positive manipulation, namely, enriched environment (EE), on the depressive-like behavior observed in SERT^−/−^ rats. We found that one month of EE exposure normalized the anhedonic and anxious-like phenotype characteristics of this animal model. Moreover, we observed that EE exposure also restored the molecular alterations in the prefrontal cortex by positively modulating the expression of the neurotrophin Bdnf, and of spines and gamma-aminobutyric acid (GABA)ergic markers. Overall, our data confirm the depression-like phenotype of SERT^−/−^ rats and highlight the ability of EE to restore behavioral and molecular alterations, thus promoting the opportunity to use EE as a supporting non-pharmacological approach to treat mood disorders.

## 1. Introduction

The world perception is extremely subjective and greatly depends on different factors, such as the genetic background of each person, life experiences, and the environment we are exposed to. 

This concept is particularly true for psychiatric pathologies that have a multifactorial etiology: the presence of genetic modifications, together with negative environmental stimuli leads to the manifestation of symptoms of mood disorders [1].

Among other genetic alterations, the serotonin transporter (5-HTT in humans, SERT in rodents), a crucial component in the control of the serotonergic tone, is characterized by the presence of a functional polymorphism. In particular, people with the short allelic variant of the polymorphic region of the serotonin transporter (5-HTTLPR) show impairments in 5-HTT gene transcription compared to those carrying the long allele, and deficits in coping with negative situations [2,3], such as childhood trauma [4]. Accordingly, associations between the 5-HTTLPR short allele and increased risk for depression have been found [4,5,6,7,8,9,10]. Conversely, when exposed to supportive environmental stimuli, short allele carriers benefit most [11,12,13]. A substantial body of evidence has demonstrated that a positive environment can protect against the effects of negative situations and reduce the risk for depression, particularly in early life, but also in adulthood [14,15,16,17,18]. This is in line with the recent theory of differential susceptibility, arguing that short allele carriers have an increased sensibility to the external conditions with worst outcomes after facing negative conditions, but also best effects after supportive conditions [14,19].

The 5-HTTLPR short allele is modeled in rodents by deleting the SERT gene, thus allowing a deeper study of the lifelong consequences of SERT malfunctioning on behavior and underlying brain circuits. At the basal level, SERT^−/−^ rats show depressive-like symptoms, such as anhedonia, social deficits, and anxiety [3,20,21]. Furthermore, SERT knockout rats behaviorally and neurally mimic stress sensitivity as found in 5-HTTLPR short allele carriers [22]. Moreover, at the molecular level, we demonstrated that SERT deletion induced impairments in neuroplastic mechanisms involving Brain Derived Neurotrophic Factor (Bdnf), as well as a reduction in the spine and gamma-aminobutyric acid (GABA)ergic markers not only in adulthood, but also during the development [23,24,25,26]. Interestingly, as observed in human 5-HTTLPR short allele carriers, SERT knockout rats are also more responsive to positive environmental stimuli, for example, to tactile stimulation in early life [27], and conditioned reward in adulthood [28]. 

Unfortunately, first-line antidepressant treatment is only partially effective in treating mood disorders [29], possibly because the target of first-line antidepressant treatment, the 5-HTT, is reduced in those vulnerable to depression [30,31]. Therefore, non-pharmacological therapies are emerging [32,33], which can be used concurrently with pharmacotherapy to improve treatment outcomes. Interestingly, these alternative approaches can also be mimicked at the preclinical level by housing the animal in an enriched environment (EE), resembling psychological support [34,35,36]. Because individuals characterized by an inherited reduction in SERT expression are more sensitive to both negative and positive environmental stimuli [37], we hypothesized that SERT knockout rats would benefit most from EE and would display a reduction in depression-like behavior and related neuroplasticity markers when compared to wild-type animals. 

Accordingly, it has previously been demonstrated that EE in adulthood reduced anxiety- but not depression-like behavior in SERT knockout mice [38]. Here we replicated and extended this study in SERT knockout rats. Specifically, we exposed SERT^+/+^ and SERT^−/−^ male rats to a normal environment (NE) defined as paired housing in standard cages with limited enrichment, or to EE consisting of groups of 10 animals housed in large cages with additional toys, tunnels, and shelters for one month. During this month, the animals were subjected to behavioral tests measuring depression- and anxiety-like behavior. After NE/EE exposure and behavioral testing, the SERT knockout animals and their controls were sacrificed to evaluate the effects of environmental manipulation on the expression of Bdnf, GABAergic, and spines markers in the prefrontal cortex (PFC), a brain region that is highly responsive to environmental stimuli and adaptive behavioral responses [39,40].

## 2. Materials and Methods 

### 2.1. Animals

SERT^−/−^ (Slc6a41Hubr) rats were obtained through N-ethyl-N-nitrosourea (ENU) mutagenesis, as previously described [41]. 

SERT^−/−^ and SERT^+/+^ rats were housed under standardized conditions involving a 12/12-h light/dark cycle, at 22 °C and around 80% of humidity with access *ad libitum* to food and water. 

All experimental procedures were approved by the Central Committee on Animal Experiments (Centrale Commissie Dierproeven, CCD, The Hague, The Netherlands – ethic approval code: AVD1030020187064), limiting the number of animals used and minimizing animal suffering. 

### 2.2. Housing Conditions

After paired housing conditions from weaning, at adulthood (postnatal day 140), SERT^−/−^ and SERT^+/+^ male rats (n = 40) were assigned to either of the two experimental groups and housed in a normal or enriched cage in a random way. NE involved paired housing in a cage sized 42.5 × 26.6 × 18.5 cm enriched with a rat retreat, while EE consisted of groups of 10 animals housed in cages sized 100 × 54.5 ± 48 cm with cage enrichment that included toys, tunnels, and nesting places of different colors and textures to promote exploration behavior. Both NE and EE experimental groups were housed in the same room, and all the animals were handled once a week.

### 2.3. Behavioral Procedures

**Figure 1:**
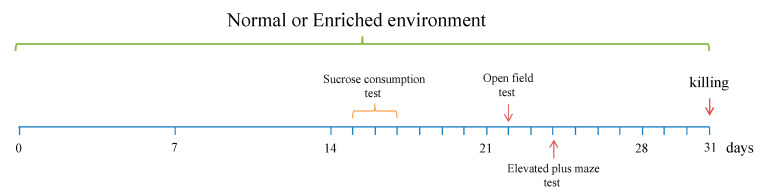
Experimental paradigm. The sucrose consumption test was performed at day 15 to 17, the open field test were conducted at day 22 and the elevated plus maze test at day 25 after the start of the EE. At day 31, animals were killed for the molecular analyses.

From day 15 to day 24 of the housing procedure, we performed behavioral tests following the schedule represented in Figure 1. 

### 2.4. Sucrose Consumption

Anhedonia, a core feature of depression, was investigated by the sucrose consumption test, and the anhedonic-like behavior refers to a reduction of the preference for the sucrose solution in the sucrose consumption test [42]. During this procedure, animals were single-housed for 4 h a day (for three days—two of habitation and one of the test), and they had free access to two bottles: one filled with water and one of 4% sucrose solution. Fluid consumption (g) was measured weighing the bottles before and 4 h after the start of the test, and the measures were used to calculate sucrose preference (sucrose intake in ml divided by total intake × 100%).

### 2.5. Open Field

Taking advantage of the natural predisposition of rodents to explore new environments and spaces and to assess novelty-induced locomotor activity, we exposed the animals to the open field test by placing each rat in a squared arena (1 m × 1 m) and by letting them free to move and explore the novel environment for 5 min. In particular, rats were placed in the center of the arena and were recorded. The distance moved, as well as the velocity, were measured using EthoVision XT (version 3.1., Noldus) software (Noldus, Wageningen, The Netherlands).

### 2.6. Elevated Plus-Maze (EPM)

Considering the innate fear of rodents for open and elevated spaces, we employed the EPM test to evaluate the anxiety-like behavior of the animals. In particular, a plus-shaped platform was located at 50 cm from the floor. The maze consisted of two closed arms (50 cm × 10 cm with 40 cm high walls) and two open arms of the same dimensions and without walls. The two equal arms were positioned opposite each other, and the four arms formed a central platform at their intersection. At the beginning of the test, each rat was placed on the central platform looking at one of the open arms. The position and the movements of the animals were recorded for 5 min, and the time spent in the closed arms, in the center, and in the open arms of the arena were analyzed using EthoVision XT (version 3.1., Noldus) software. 

### 2.7. Brain Tissue Collection

At the end of the 31st day, animals were decapitated. To perform the molecular analyses, the PFC was dissected according to the atlas of Paxinos and Watson [43] from slices of 2-mm-thickness (plates 6–9, including Cg1, Cg3, and IL sub-regions), frozen on dry ice and stored at −80 °C. 

### 2.8. RNA Extraction and Quantitative Real-Time PCR Gene Expression Analysis

Total RNA was isolated, as previously described [44]. The samples were subsequently processed for real-time polymerase chain reaction (RT-PCR) to assess the expression of total *Bdnf*, *Bdnf* long 3′UTR, Post Synaptic Density Protein 95 (*Psd95*), Cell Division Cycle 42 (*Cdc42*), Glutamate Decarboxylase 65 (*Gad65*), Glutamate Decarboxylase 67 (*Gad67*), GABA vesicular transporter (*Vgat*), Parvalbumin (*Pvalb*) and GABA Type A Receptor Subunit Alpha2 (*GABA_A_γ2*). Primer and probes sequences are specified in Table 1, and all the samples were run in a 384 well plate with *36b4* as normalizing internal control and in triplicate. 

After 10 min of incubation at 50 °C for the RNA retrotranscription, and 5 min at 95 °C for the TaqMan polymerase activation, 39 cycles of PCR (10 s at 95 °C to allow the melting process and 30 s at 60 °C for the annealing and extension) were performed. To calculate the relative expression of each gene, a comparative cycle threshold (Ct) method was used.

### 2.9. Protein Extraction and Western Blot Analysis

To investigate mature BDNF (mBDNF), PSD95, CDC42 protein levels in the membrane fraction, and GAD65 and GAD67 protein levels in the whole homogenate of the PFC, we employed Western blot analyses. 

Protein extraction and quantification were performed as previously described [45].

Equal amounts of protein of each sample were run on 10% SDS-polyacrylamide gels under reducing conditions and then electrophoretically transferred on PVDF membranes (GE Healthcare Life Sciences). Blots were blocked with 5% non-fat milk to cover non-specific bindings and then incubated with primary and secondary antibodies (details are specified in Table 2). The Western Lightning Clarity ECL (Bio-Rad Laboratories, Segrate, Italy) and the Chemidoc MP imaging system (Bio-Rad Laboratories, Segrate, Italy) was used to visualize the immunocomplexes. Finally, after normalization of the β-ACTIN, protein levels were quantified, evaluating the band densities (ImageLab, Bio-Rad Laboratories, Segrate, Italy).

### 2.10. Statistical Analysis

Statistical analyses were performed with the “IBM SPSS Statistics, version 24” software (IBM, Segrate, Italy), and two-way ANOVA followed by Fisher’s PLSD was employed to analyze the results. Significance was assumed for *p* < 0.05, and data are graphically presented as means ± standard error (SEM).

All the details of the statistical results are listed in Supplementary Table S1. 

## 3. Results

### 3.1. One Month of EE Normalized the Depression- and Anxiety-Like Behavior in SERT^−/−^ Rats

**Figure 2:**
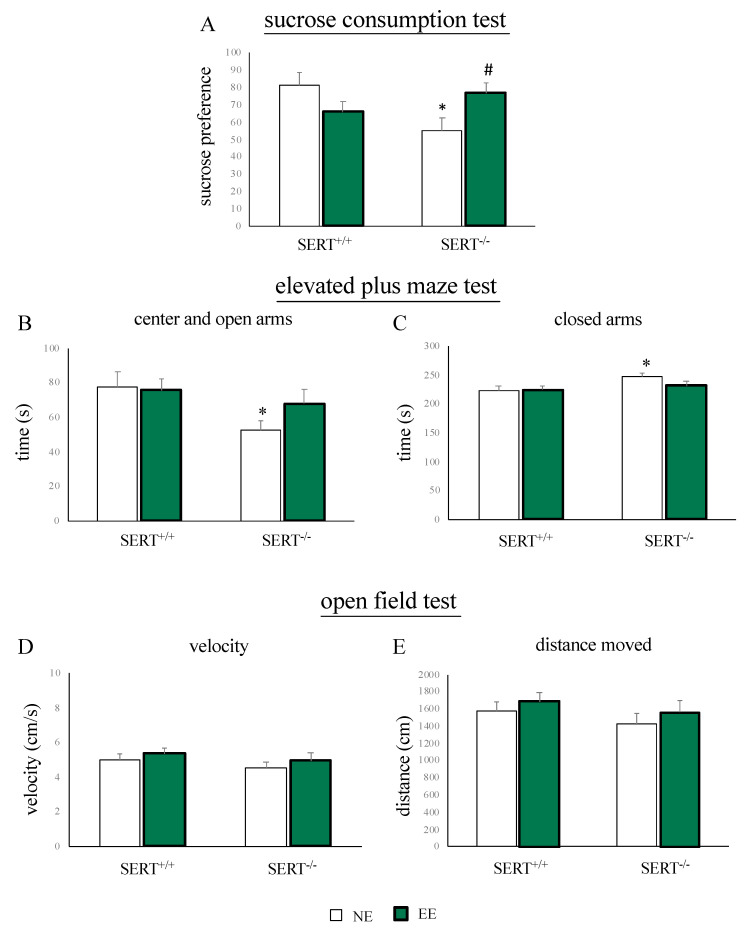
Behavioral characterization of SERT^+/+^ and SERT^−/−^ rats subjected to one month of exposure to NE (normal environment) or EE (environmental enrichment). (**A**) Sucrose preference in the sucrose consumption test. (**B**) Time spent in the center and in the open arms (**C**) and in the closed arms during the EPM test. (**D**) Velocity in the open field test. (**E**) Distance moved in the open field test. The data are presented as mean ± standard error of the mean (SEM). * *p* < 0.05 vs. SERT^+/+^/NE; # *p* < 0.05 vs. SERT^−/−^/NE; two-way analysis of variance (ANOVA) followed by protected least significant difference (PLSD).

We previously demonstrated that SERT^−/−^ rats display anhedonia and anxiety-like behavior under standard housing conditions [21]. Here we tested whether these phenotypes could be normalized by EE. The sucrose consumption test was used to measure anhedonia. As shown in Figure 2A, we found a significant reduction in sucrose preference in SERT^−/−^ rats versus controls under NE conditions (−26% *p* < 0.05 vs. SERT^+/+^/NE). This phenotype, indicative for anhedonia, was normalized by EE (+22% *p* < 0.05 vs. SERT^−/−^/NE). 

To measure anxiety-like behavior, we subjected the animals to the EPM test. We observed that, compared to wild-type controls, SERT^−/−^ rats, spent less time in the center and in the open arms (−25 s *p* < 0.05 vs. SERT^−/−^/NE) and more time in the closed arms (+25 s *p* < 0.05 vs. SERT^−/−^/NE) of the EPM. Interestingly, these alterations in the time spent in the different places of the maze were not present in SERT^−/−^ animals exposed to the EE (open and center: +16 s *p* > 0.05 vs. SERT^−/−^/NE; closed: −16 s *p* > 0.05 vs. SERT^−/−^/NE) (Figure 2B,C). 

Finally, novelty-induced locomotor activity was assessed using the open field test. No changes in activity were observed, neither in genotype groups nor in differential housing groups (Figure 2D,E).

### 3.2. The EE Improved Neuroplastic Mechanisms in SERT^−/−^ Rats 

Since we previously found that SERT^−/−^ rats display impairments in the expression of the neuroplasticity marker Bdnf in the prefrontal cortex, both at mRNA and protein level [24], we evaluated if EE would alter Bdnf transcription and translation. 

**Figure 3:**
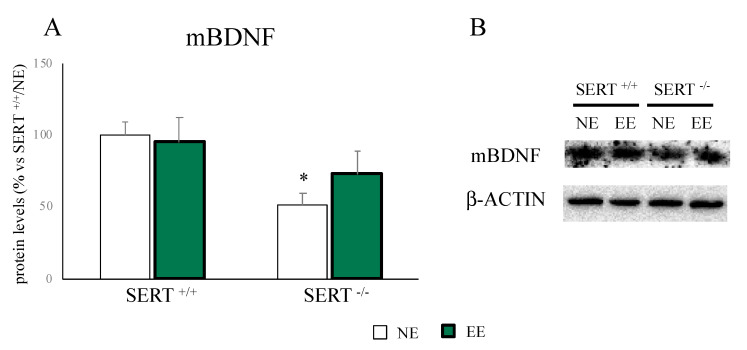
Analyses of mBDNF protein levels in the PFC of SERT^+/+^ and SERT^−/−^ rats subjected to one month of exposure to NE (normal environment) or EE (environmental enrichment) (**A**) and its representative western blot bands (**B**). The data are presented as percent change of SERT^+/+^/NE and are expressed as mean ± standard error of the mean (SEM). * *p* < 0.05, vs. SERT^+/+^/NE; two-way ANOVA with PLSD.

As shown in Figure 3A,B, the significant reduction in mBDNF protein levels found in SERT^−/−^ rats (−48% *p* < 0.05 vs. SERT^+/+^/NE) was partially restored by the EE (+41% *p* > 0.05 vs. SERT^−/−^/NE). 

In line, we found a similar effect at the transcriptional level (Table 3). Indeed, we observed a slight reduction in the transcription of the *Bdnf* long pool of transcripts in SERT^−/−^ rats versus controls under normal housing conditions (−20% *p* > 0.05 vs. SERT^+/+^/NE) and an upregulation in EE exposed SERT^−/−^ rats (+50% *p* < 0.05 vs. SERT^−/−^/NE) (Table 3). Moreover, we found an upregulation of total *Bdnf* expression in SERT^−/−^ rats exposed to the positive housing conditions (+50% *p* < 0.05 vs. SERT^−/−^/NE) (Table 3). 

### 3.3. The Reduction of Spine Markers Expression in SERT^−/−^ Rats Is Normalized by EE

As a proxy of spine functionality, we measured the expression of some markers of dendritic spine densities to assess a possible positive effect of EE in SERT^−/−^ rats versus wild-type counterparts. 

**Figure 4:**
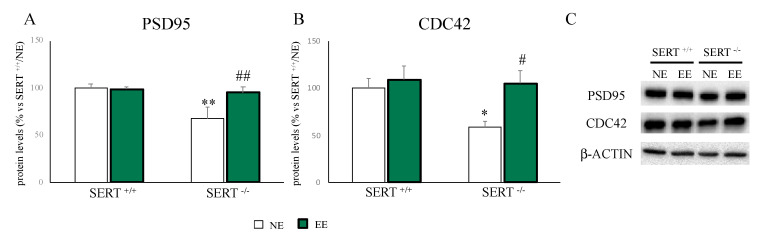
Analyses of PSD95 (**A**) and CDC42 (**B**) protein levels in PFC of SERT^+/+^ and SERT^−/−^ rats subjected to one month of exposure to NE (normal environment) or EE (environmental enrichment) and their representative western blot bands (**C**). The data are presented as percent change of SERT^+/+^/NE and are expressed as mean ± standard error of the mean (SEM). * *p* < 0.05, ** *p* < 0.01 vs. SERT^+/+^/NE; # *p* < 0.05, ## *p* < 0.01 vs. SERT^−/−^/NE two-way ANOVA with PLSD.

As shown in Figure 4, we found a downregulation in PSD95 (Figure 4A–C) and CDC42 (Figure 4B,C) protein levels (PSD95: −32% *p* < 0.01 vs. SERT^+/+^/NE; CDC42: −42% *p* < 0.01 vs. SERT^+/+^/NE) in SERT^−/−^ rats compared to wild-type rats, while EE normalized their PSD95 levels (PSD95: (+40% *p* < 0.01 vs. SERT^−/−^/NE; CDC42: +80% *p* < 0.01 vs. SERT^−/−^/NE). 

In line with the protein expression data, mRNA expression levels of *Psd95* were significantly reduced in SERT^−/−^ rats (−15% *p* < 0.05 vs. SERT^+/+^/NE) and upregulated by EE exposure (+23% *p* < 0.01 vs. SERT^−/−^/NE). Similarly, we found a slight reduction in *Cdc42* mRNA levels in SERT^−/−^ rats (−9% *p* > 0.05 vs. SERT^+/+^/NE) and a trend to an increase in SERT^−/−^ rats exposed with EE (+20% *p* > 0.05 vs. SERT^−/−^/NE) (Table 3). 

### 3.4. GABAergic System Alterations of SERT^−/−^ Rats Are Restored by the EE

**Figure 5:**
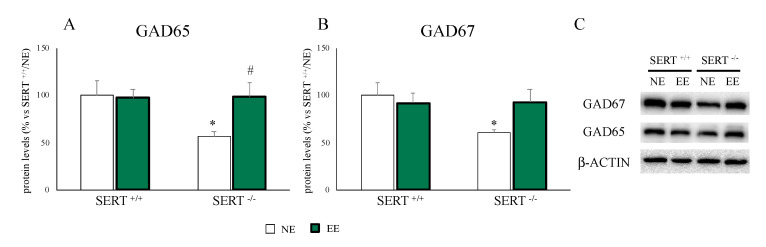
Analyses of GAD65 (**A**) and GAD67 (**B**) protein levels in the PFC of SERT^+/+^ and SERT^−/−^ rats subjected to one month of exposure to NE (normal environment) or EE (environmental enrichment) and their representative western blot bands (**C**). The data are presented as percent change of SERT^+/+^/NE and are expressed as mean ± standard error of the mean (SEM). * *p* < 0.05, vs. SERT^+/+^/NE; # *p* < 0.05, vs. SERT^−/−^/NE two-way ANOVA with PLSD.

The major inhibitory neurotransmitter in the brain is GABA, and alterations in its system are often linked to anxiety [46]. Given the normalization of anxiety-like behavior in EE versus NE exposed SERT^−/−^ animals, we analyzed the expression levels of GAD65 and GAD67, which are responsible for the production of GABA in the brain. As shown in Figure 5, we found a downregulation of both GAD65 and GAD67 in SERT^−/−^ rats (GAD65: −43% *p* < 0.05 vs. SERT^+/+^/NE; GAD67: −39% *p* < 0.05 vs. SERT^+/+^/NE) which was normalized by EE (GAD65: +87% *p* < 0.05 vs. SERT^−/−^/NE; +53% *p* > 0.05 vs. SERT^−/−^/NE). 

In line, the mRNA levels of *Gad65* and *Gad67* were downregulated in SERT^−/−^ rats (*Gad65*: −21% *p* < 0.05 vs. SERT^+/+^/NE; *Gad67*: −19% *p* < 0.05 vs. SERT^+/+^/NE) and these changes were normalized by the EE (*Gad65*: +39% *p* < 0.01 vs. SERT^−/−^/NE; *Gad67*: +15% *p* > 0.05 vs. SERT^−/−^/NE). Moreover, we found an increase in *Gad67* expression in SERT^+/+^ animals with EE (+21% *p* < 0.05 vs. SERT^+/+^/NE) (Table 3). 

Finally, as shown in Table 3, we found in SERT^−/−^ rats compared to wild-type controls a downregulation in the expression of the vesicular GABA transporter *Vgat*, the receptor subunit *GABA_A_γ2,* and of the GABAergic interneurons marker *Pvalb (Vgat:* −33% *p* < 0.05 vs. SERT^+/+^/NE; *GABA_A_γ2*: −17% *p* < 0.05 vs. SERT^+/+^/NE; *Pvalb*: −20% *p* < 0.05 vs. SERT^+/+^/NE). These down-regulations were normalized by EE (*Vgat*: +13% *p* > 0.05 vs. SERT^−/−^/NE; *GABA_A_γ2:* +20% *p* < 0.05 vs. SERT^−/−^/NE; *Pvalb:* +34% *p* < 0.01 vs. SERT^−/−^/NE). 

## 4. Discussion

SERT^−/−^ rats are one of the most employed animal models to study mechanisms related to vulnerability to depression, since they display different features of the human illness both at a behavioral and molecular level [21,23,24,25,47,48].

By using this animal model, we here confirm the depression- and anxiety-like phenotypes of SERT^−/−^ rats, as well as the impairments, at molecular level, of the neurotrophic factor Bdnf, and the spine and GABAergic markers [23,24,25]. Interestingly, we observed that one month of EE exposure normalized these alterations both at a behavioral and molecular level. These findings confirm our hypothesis that deleting the SERT induces an increased sensibility to the external environment supporting the idea that a stimulating situation can have a beneficial impact on SERT^−/−^ rats.

In the sucrose consumption test, we found a reduced preference of the sucrose solution relative to plain water, confirming the anhedonic phenotype of this animal model [21]. Similarly, in line with the literature results [21,49], we found anxiety-like behavior in SERT^−/−^ rats tested in the EPM test. Interestingly, one month of EE normalized these anxiety and depressive-like symptoms in SERT^−/−^ rats. Accordingly, it has been shown that exposure to positive stimuli, such as the EE or physical exercise, normalized the pathological phenotype in different animal models of depression [34,50,51] suggesting that a constructive environment might help in ameliorating human traits of mood disorders. Notably, we found a specific effect of the EE on SERT^−/−^ rats, while no behavioral improvements in SERT^+/+^_,_ in line with the major sensitivity for external stimuli when SERT functionality is reduced [37].

Seen the restorative effect of the EE at a behavioral level, we decided to deepen the molecular mechanisms, possibly underlying the positive outcome of EE. In particular, since we previously demonstrated that SERT^−/−^ rats are characterized by an impairment in neuroplastic mechanisms [24], we measured Bdnf transcription and translation. In line with our previous results [24], we found reduced mBDNF protein levels, as well as of the pool of the long transcripts of Bdnf in SERT^−/−^ rats with a normalization, due to EE exposure. This perfectly fits the increase in neurotrophin levels after positive environmental stimuli that are often paralleled by improvements at a behavioral level [39,52].

One of the roles of Bdnf includes the promotion of spine maturation and formation [53,54,55], for instance, in animal models of depression [25,56,57]. Here, along with our previous data [25], we found a reduction in the spine markers PSD95 and CDC42 in NE exposed SERT^−/−^ rats. Like for Bdnf, one month of EE was able to normalize their expression. Interestingly, it has been demonstrated that antidepressant treatments normalized spine atrophy and density reduction in animal models of depression [58,59], suggesting that also EE, by increasing these spine markers expression, could have a restorative effect on spine morphology.

Finally, given the tight co-play between the GABA system and anxious behavior [46], we analyzed the expression of GABAergic markers previously found to be impaired in SERT^−/−^ rats [23]. Interestingly, we found a reduction in the expression of GAD65 and GAD67, enzymes responsible for the production of the inhibitory neurotransmitter, as well as of the vesicular transporter *Vgat*. Moreover, in SERT^−/−^ animals, we found a reduction in *Pvalb*, a GABAergic interneurons marker [60], as well as in *GABA_A_γ2*, the most abundant GABA receptor subunit in the adult brain which deletion promotes an anxiety-like behavior in rodents [61,62]. Furthermore, in line with the results obtained from the EPM test, all these GABAergic alterations were normalized by EE, further supporting the positive impact of enriched housing in SERT^−/−^ rats.

Taken the behavioral and molecular data together, we observed a specific effect of EE on SERT^−/−^ rats, while SERT^+/+^ animals seemed to be unaffected by the environmental manipulation. This is in line with the vantage sensitivity theory stating that some plasticity factors, like the 5-HTTLPR s-allele disproportionally benefit sensitive individuals [19]. Individuals not carrying such plasticity factors, on the other hand, remain largely unaffected by positive environments. The differential susceptibility theory states that individuals carrying plasticity factors are sensitive to both positive and negative stimuli [14]. As mentioned before, there is extensive evidence that the SERT gene behaves according to this latter theory. However, a limitation of the present study is that we did not expose the animals to a negative environment, like social isolation, to investigate whether behavioral and molecular parameters would worsen compared to the normal housing condition. Moreover, seen the different impact of the EE in male and female and the prevalence of the pathology in women [63,64], we think that further studies employing female rats could improve our results and highlight possible differences in between the two sexes at basal level and in response to the positive stimuli.

## 5. Conclusions

In summary, our data confirms that the behavioral and molecular characterization of SERT^−/−^ rats at basal level. Moreover, it also provides new interesting insights on the possible use of non-pharmacological approaches to be employed as supportive therapies to treat psychopathologies, improving patients’ compliance, and increasing the successful treatment rate.

## Figures and Tables

**Table 1:** Forward and reverse primers and probes sequences used in real-time polymerase chain reaction (RT-PCR) analyses, acquired from Eurofins MWG-Operon or from Life Technologies, which does not reveal the sequences.

Gene	Forward Primer	Reverse Primer	Probe
Total *Bdnf*	AAGTCTGCATTACATTCCTCGA	GTTTTCTGAAAGAGGGACAGTTTAT	TGTGGTTTGTTGCCGTTGCCAAG
*Psd95*	CAAGAAATACCGCTACCAAGATG	CCCTCTGTTCCATTCACCTG	TCAACACGGACACCCTAGAAGCC
*Cdc42*	AAGGCTGTCAAGTATGTGGAG	GCTCTGGAGATGCGTTCATAG	CCTGCGGCTCTTCTTCGGTTCT
*Gad65*	TGAGGGAAATCATTGGCTGG	TCCCCTTTTCCTTGACTTCTG	TGCCATCTCCAACATGTACGCCA
*Gad67*	ATACTTGGTGTGGCGTAGC	AGGAAAGCAGGTTCTTGGAG	AAAACTGGGCCTGAAGATCTGTGGT
*Vgat*	ACGACAAACCCAAGATCACG	GTAGACCCAGCACGAACATG	TTCCAGCCCGCTTCCCACG
*GABA_A_Υ2*	ACTCATTGTGGTTCTGTCCTG	GCTGTGACATAGGAGACCTTG	ATGGTGCTGAGAGTGGTCATCGTC
*Pvalb*	CTGGACAAAGACAAAAGTGGC	GACAAGTCTCTGGCATCTGAG	CCTTCAGAATGGACCCCAGCTCA
*36b4*	TCAGTGCCTCACTCCATCAT	AGGAAGGCCTTGACCTTTTC	TGGATACAAAAGGGTCCTGG
Gene	Accession number		assay ID
*Bdnf* long 3’UTR	EF125675		Rn02531967_s1

**Table 2:** Antibodies condition used in the western blot analyses.

	Primary Antibody	Secondary Antibody
mBDNF (Icosagen)	1:1000 Bovin Serum Albumin (BSA) 5%	Anti-mouse 1:1000 Milk3%
	Over/Night (O/N) 4 °C	1 h Room Temperature (RT)
PSD95 (Cell Signalling)	1:4000 BSA 5% O/N 4 °C	Anti-rabbit 1:8000 Milk3% 1 h RT
CDC42 (Cell Signalling)	1:1000 BSA5% O/N 4 °C	Anti-rabbit 1:1000 Milk3% 1 h RT
GAD65 (Millipore)	1:2000 Milk5% O/N 4 °C	Anti-rabbit 1:1000 Milk3% 1 h RT
GAD67 (AbCAM)	1:2500 Milk3% O/N 4 °C	Anti-mouse 1:5000 Milk3% 1 h RT

**Table 3:** Analyses of *Bdnf*, spine, and GABAergic markers mRNA levels in the PFC of SERT^+/+^ and SERT^−/−^ rats subjected to one month of exposure to NE or EE. The data are presented as percent change of SERT^+/+^/NE and are expressed as mean ± standard error of the mean (SEM). * *p* < 0.05 vs. SERT^+/+^/NE; # *p* < 0.05, ## *p* < 0.01, ### *p* < 0.001 vs. SERT^−/−^/NE two-way ANOVA with PLSD.

Gene	SERT^+/+^/NE	SERT^+/+^/EE	SERT^−/−^/NE	SERT^−/−^/EE
Total *Bdnf*	100 ± 3	110 ± 15	97 ± 5	146 ± 6 ###
*Bdnf* long 3’UTR	100 ± 5	143 ± 14	80 ± 6	121 ± 8 #
*Psd95*	100 ± 3	100 ± 6	85 ± 3 *	105 ± 5 ##
*Cdc42*	100 ± 4	104 ± 8	91 ± 2	110 ± 9
*Gad65*	100 ± 2	115 ± 7	79 ± 2 *	110 ± 12 ##
*Gad67*	100 ± 3	121 ± 9 *	81 ± 4 *	93 ± 10
*Vgat*	100 ± 6	113 ± 9	77 ± 3 *	88 ± 10
*GABA_A_Υ2*	100 ± 2	107 ± 6	83 ± 4 *	100 ± 6 #
*Pvalb*	100 ± 2	101 ± 9	80 ± 5 *	107 ± 6 #

## Supplementary Materials

The following are available online at https://www.mdpi.com/2073-4425/11/11/1248/s1, Table S1: statistical details of behavioral and molecular analyses.
