# Supplementary material for: Enrichment Environment Positively Influences Depression- and Anxiety-Like Behavior in Serotonin Transporter Knockout Rats through the Modulation of Neuroplasticity, Spine, and GABAergic Markers
Source: Genes (Basel). 2020 Oct 23;11(11):1248. doi: 10.3390/genes11111248 (PMC7690660; doi:10.3390/genes11111248)
Supplement: Supplementary file 1 [file genes-11-01248-s001.pdf]

Table S1. statistical details of behavioral and molecular analyses.

**Gene.**	**two-way ANOVA**
sucrose preference	genotype X EE interaction: $F_{(1,30)} = 7.463$ $p < 0.05$
time in the center and in the open arms (EPM)	genotype: $F_{(1,35)} = 4.904$ $p < 0.05$
time in the closed arms (EPM)	genotype: $F_{(1,35)} = 4.904$ $p < 0.05$
Velocity (OFT)	Not Significant
Distance moved (OFT)	Not Significant
mBDNF	genotype: $F_{(1,21)} = 6.266$ $p < 0.05$
Total *Bdnf*	EE: $F_{1,37)} = 11.227$ $p < 0.01$<br>genotype X EE interaction: $F_{(1,37)} = 4.930$ $p < 0.05$
Bdnf long 3'UTR	EE: $F_{(1,38)} = 7.927$ $p < 0.01$
PSD95	genotype: $F_{(1,38)} = 8.923$ $p < 0.01$<br>EE: $F_{(1,38)} = 4.604$ $p < 0.05$<br>genotype X EE: $F_{(1,38)} = 5.591$ $p < 0.05$
CDC42	EE: $F_{(1,38)} = 4.718$ $p < 0.05$
Psd95	EE: $F_{(1,38)} = 4.931$ $p < 0.05$;<br>genotype X EE interaction: $F_{(1,38)} = 4.641$ $p < 0.05$
Cdc42	Not Significant
GAD65	genotype X EE: $F_{(1,18)} = 5.852$ $p < 0.05$
GAD67	genotype X EE: $F_{(1,18)} = 2.919$ $p > 0.05$
Gad65	EE: $F_{(1,38)} = 10.242$ $p < 0.01$
Gad67	genotype: $F_{(1,35)} = 11.422$ $p < 0.01$<br>EE: $F_{(1,35)} = 5.767$ $p < 0.05$
Pvalb	EE: $F_{(1,37)} = 4.537$ $p < 0.05$<br>genotype X EE: $F_{(1,37)} = 3.909$ p=0.056
Vgat	genotype: $F_{(1,37)} = 10.172$ $p < 0.01$
GABA$_A$ 2	genotype: $F_{(1,38)} = 6.149$ $p < 0.05$<br>EE: $F_{(1,38)} = 5.842$ $p < 0.05$;
